# Design of Incentive Contract for Technological Innovation of New Energy Vehicles with Asymmetric Information

**DOI:** 10.3390/ijerph16224544

**Published:** 2019-11-17

**Authors:** Dong Cai, Chunxiang Guo, Yue Tan

**Affiliations:** Business School, Sichuan University, Chengdu 610065, China; caidong3@sina.com (D.C.); guochunxiang@scu.edu.cn (C.G.)

**Keywords:** asymmetric information, new energy vehicles, technological innovation, incentive contracts

## Abstract

The improvement of China’s new energy automobile technology is one of the most pressing issues for the government and manufacturers, given that the existing new energy automobile subsidy policy is about to be withdrawn completely. Considering that the manufacturer has the private information of the initial technology level of new energy vehicles, its technology can be improved by means of technological innovation. Using principal–agent and regulation theory, this paper studies how the government designs incentive contracts to motivate manufacturers to strive to upgrade new energy automotive technology. The study has obtained a quantitative incentive contract under full information and a quantitative screening contract with asymmetric information, which provides an effective reference for the design of government subsidy contracts. It was found that the existence of asymmetric information reduces the expected net utility of the government in incentive projects, and the technology upgrading of low-level manufacturers is insufficient, but will not affect the technology upgrading of high-level manufacturers who will get information rent. The conclusion has good reference value and guiding significance for government policy-making with asymmetric information.

## 1. Introduction

In order to promote the technological progress and market development of new energy vehicles, the Ministry of Industry and Information Technology of China formulated and implemented the Regulations for the Admission of New Energy Vehicle Manufacturers and Products in 2009. The Chinese Government has promulgated relevant policies to promote the development of new energy vehicles. With the support of relevant policies, the new energy strategy started in 2009–2012, and the supply chain gradually formed. New energy vehicles were developing rapidly, with new automobile manufacturers emerging continuously from 2013 to 2016. From 2017 to 2019, the number of new energy vehicles put into operation continued to grow, and the market scale continued to expand [1].

With the support of government policy, China’s new energy vehicles have achieved huge development in a short period of time, especially in terms of market development, thus achieving a stable market. However, the technological progress of new energy vehicles is very limited. According to the report of the Global Electric Vehicle Development Index 2018 issued by Roland Berger and Aachen Automotive Engineering Technology Co., Ltd., a German automotive research institute, in August 2018, China ranked among the seven major automotive countries at the technological level. Known as the penultimate, China’s new energy vehicle technology level is far from the top international level, especially in terms of core battery technology. In September 2018, the *Guidelines for Evaluating the Development Effect and Technical Policy of China’s New Energy Vehicles*, issued by the China Automobile Technology Research Center affiliated with the state-owned Assets and Management Commission of the State Council, also pointed out that there were obvious gaps between China’s new energy vehicles and foreign countries in intelligent key technologies, fuel cell technology, and so on.

The existing subsidy policies have achieved remarkable results in promoting the development of new energy market, but the effect in promoting the technological progress of new energy vehicles is not ideal. The main reason for this is that it cannot effectively solve the problem of asymmetric information between new energy automobile manufacturers and the government. The incentives for manufacturers to upgrade their technology are not targeted. Instead, it has caused a series of “defrauding government subsidies” incidents. For example, on 8 September 2016, the ministry of finance revealed that five new energy automobile manufacturers, including the Suzhou Jimsey Bus Manufacturing Co., Ltd., intended to defraud the state of financial subsidies exceeding 1 billion yuan. On 7 February 2017, the Ministry of Industry and Information Technology issued a new ticket for new energy automobile fraudulent compensation enterprises, and announced the administrative penalty decisions for seven fraudulent repair enterprises, including Jinhua Youth Automobile and Chongqing Lifan. The drawbacks of the existing subsidy policy have also caused tremendous economic losses to the country.

To this end, Miao Wei, Minister of Industry and Information Technology, at the 2008 China Electric Vehicle 100 People’s Congress Forum, said that in the case of gradual withdrawal of the subsidy policy, the follow-up policy needs to be studied in advance, and the layout should be grasped. At the same time, it emphasizes the need for further breakthroughs in the core technology of new energy vehicles, and relevant supporting policies need to be developed urgently. Considering the practical problems and the requirements of the national strategic layout, it is urgent to determine a subsidy policy that can effectively improve the technology of new energy vehicles after the current subsidy policy has declined, especially to effectively solve the problem of information asymmetry in the subsidy policy.

At present, the research of domestic and foreign scholars on new energy automobile subsidy policy mainly focuses on the influence of government subsidies on market development. For example, some scholars have analyzed China’s new energy vehicle policy in recent years, and revealed how these policies were systematically linked to support and guide the rapid development of new energy automobiles in China [2,3,4,5]. Some scholars constructed a model of government incentives for sales efforts for new energy automobile sales enterprises with behavioral externalities through subsidies, and studied the impact of government subsidies, the sales objectives of subsidies, and other factors on system performance [6,7,8,9,10]. Gass et al. [11] considered that preferential price and tax relief policies provided guarantees for the early development of electric vehicles. Yuan et al. [12] gave a comprehensive review of China’s policy framework for new energy vehicles. The analysis shows that policy guidance and planning play a vital role in the development of the new energy automobile industry. In order to meet the growing demand for new energy vehicles, it is necessary to speed up the construction of supporting facilities and infrastructure. Previous literature shows that with the support of relevant policies, remarkable results have been achieved in terms of market cultivation for new energy vehicles. However, due to the limitations of domestic subsidy policies and limited technical promotion, further development of the new energy vehicle market has been limited. Therefore, in the context of the imminent decline of government subsidies aimed at cultivating the market, the government focuses on using the subsidy policy to improve the technical level of new energy vehicles, which is the difference between the content of this paper and the aforementioned literature.

Some scholars have also discussed how the government should correctly guide the upgrading of technology, e.g., some scholars proposed that a combination of R&D subsidy policy and double integral policy could better improve the market mechanisms, promote technological innovation, and bring about the healthy development of industry [13,14,15,16]. Some scholars proposed that China should increase policy supply for basic R&D of the new energy automobile industry, increase funding, and encourage enterprises to independently develop and innovate technology, so as to enhance industrial competitiveness and occupy the new technology line [17,18]. Furthermore, some scholars hypothesized that with the adjustment and decline of two typical policies, the state gradually releases market signals, showing that the form and content of policy support are changing, especially in the field of industrial technology innovation and business model innovation [19,20,21]. The literature [13,14,15,16,17,18] mainly gives policy recommendations for technological innovation based upon symmetrical information. The literature [19,20,21] qualitatively analyses which policies the government should use to stimulate technological innovation in enterprises. Previous literature has shown that with the support of relevant policies, the cultivation of the new energy vehicles market has achieved remarkable results. However, due to the technical bottleneck of domestic new energy vehicles, further development of the new energy vehicle market is limited. The technical progress of new energy vehicles needs the support of government policy.

According to the principal–agent theory, the government uses the contract menu to identify the manufacturer’s private information [22]. Shen et al. [23] used procurement contracts to reveal supplier information about supply-chain risk. Chaturvedi et al. [24] also used the contract menu to design the procurement auction mechanism. The menu contract is a common form of contract, in reality. For example, all kinds of packages made by China Mobile for 4G products and salary contracts made by companies for salesmen with different risk aversion types belong to menu contracts. Because the manufacturer’s efforts are not visible, the design of incentive contract menu needs to consider adverse selection at the same time. Laffont and Tirole [25] were the first to consider adverse selection. They regulate monopolists by observing the cost. Section 6.3 of Contract Theory by Bolton and Dewatripont specifically discusses this issue and references a series of publications [22]. In recent research, Huang et al. [26] considered that suppliers have private information about initial reliability, and manufacturer process improvement can improve initial reliability. Using principal–agent theory, the optimal contract design under joint optimization of manufacturer process improvement and purchasing strategy was studied. These articles all examined the question of how to design contracts to expose the private information of agents and motivate their behavior. Therefore, this method is suitable for the government to apply in the design of incentive contracts.

To sum up, on the premise that the technology level of new energy vehicles can be improved, this paper uses the principal–agent theory to study the design of government incentive contracts under the condition of asymmetric information of the initial technology level of manufacturers. In this mechanism, the government is the principal and the manufacturer is the agent. The government first designs a set of contract menus. After the manufacturer observes the contract, it chooses according to its private information and makes efforts according to the contract requirements. In the model analysis, firstly, the contract design of the government in the ideal state of symmetric information is studied; then, the contract design of the government in asymmetric information is studied. Finally, the value of information is analyzed from the perspective of the government and manufacturers.

The remainder of this paper is organized as follows. Section 2 provides a survey of the related research and describes our model variables and assumptions. In Section 3, the incentive contract model of the government is developed and analyzed. In Section 4, the information value is analyzed with an example. The final section concludes the paper. All mathematical proof is provided in Appendix A, Appendix B, Appendix C, Appendix D and Appendix E.

The subsidy policy for new energy vehicle technology innovation is a relatively new research topic, focusing on the validity of qualitative analysis policies under symmetric information conditions [16,17,18,19,20,21,22,23,24,25,26]. This paper focuses on the initial technical level of manufacturers for symmetric information, and examines the question of how government-designed, quantitative contracts effectively encourage manufacturers to make technological innovations, as well as providing reference for government policy formulation.

## 2. Model Assumption

**Figure 1:**
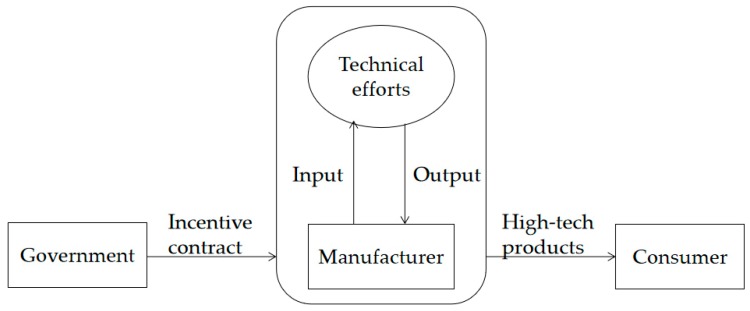
Schematic diagram of incentive system for new energy vehicle technology upgrades.

As shown in Figure 1, a system of government and individual new energy automobile manufacturers was studied. The automobile manufacturer is responsible for the research and development, production, and sales of new energy vehicles. The government designs contracts to motivate manufacturers to improve their technology. The manufacturer has the private information of the initial technological level, and the government can only observe the technological level of the products a posteriori.

It is assumed that there are two types of manufacturers in the market: those with a high initial technology level (high type: *H*) and those with a low initial technology level (low type: *L*). The high initial technology level parameter is indicated as $\beta_{H_{0}}$, while the low initial technology level parameter is indicated as $\beta_{L_{0}}$. For the convenience of the following description, it is assumed that the larger parameters represent the lower level of technology, that is, $\beta_{L_{0}}>\beta_{H_{0}}>0$. The probability of the existence of a high-level manufacturer is α (1−α), α∈(0,1). This summarizes the public knowledge of the government and the manufacturer. The government designs a set of contract menus $\left\{t_{i},\Delta \beta_{i}\right\}$ (i=H or L), without knowing the type of manufacturer. $t_{i}$ is the transfer payment in advance, and $\Delta \beta_{i}$ is the technology upgrade of manufacturer type i. The linear relationship between effort degree and technical level is indicated as $\Delta \beta_{i}=ke_{i}$, where, k is the constant and $e_{i}$ is the degree of effort for type i manufacturer to upgrade their technology [27].

**Figure 2:**
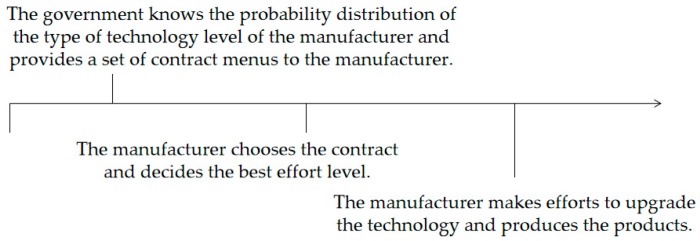
Sequence of events.

Figure 2 depicts a sequence of events: (1) The manufacturer knows his/her true technology level, but the government does not; (2) The government knows the probability distribution of the technology level of the manufacturer and provides a set of contract menus to the manufacturer; (3) The manufacturer chooses the contract and decides upon the best effort level; (4) The manufacturer makes efforts to upgrade the technology and manufactures the products; (5) The manufacturer sells the products with improved technology.

**Assumption** **1.** *The manufacturer is facing a stable market. Efforts to upgrade technology under government incentives will not lead to an increase in sales, but the improvement of technology can effectively maintain the existing stable market.*

**Assumption** **2.** *Sales prices can be observed by the government, which does not allow manufacturers to pass on the negative effects of their efforts to consumers by raising prices.*

**Assumption** **3.** *The government motivates manufacturers to make technological innovation as a public welfare project, focusing on the environmental and social effects of the technological upgrade of new energy vehicles. Environmental utility is mainly embodied by two aspects: resource saving and carbon emission reduction. Social utility is mainly embodied in social welfare. This article refers to the value of technology upgrades to consumers. The expected utility of the government is:*

(1) $$W\left(e_{i}\right)=U_{S}(e_{i})+U_{C}(e_{i})$$

In Formula (1), $U_{S}(e_{i})$ is the environmental utility brought about by upgrading technology after type i manufacturer makes effort $e_{i}$, $U_{S}(e_{i})=vs(e_{i})$; $s(e_{i})$ is the amount of energy saving and emission reduction brought about by type i manufacturer upgrading its technology, $s(e_{i})=ake_{i}$ where, a is a constant. v indicates the value coefficient of the amount of energy saving and emission reduction per unit to the government [28]. $U_{C}(e_{i})$ is the social utility brought about by upgrading technology after type i manufacturer makes effort $e_{i}$, i.e., the value brought about by technology upgrades to consumers, $U_{C}(e_{i})=bke_{i}$ where, b is a constant.

**Assumption** **4.** *The manufacturer is a rational person and only pays attention to his/her own economic utility. If the profit obtained after accepting the incentive contract is less than that obtained by not accepting the incentive contract, the manufacturer has the right to refuse the contract.*

(2) $$\pi_{i_{0}}=t_{i}-\frac{1}{2}n\beta_{i_{0}}e_{i}{}^{2}\geq 0$$

In Formula (2), $\pi_{i_{0}}$ is the retention utility of type i manufacturer. If $t_{i}-\frac{1}{2}n\beta_{i_{0}}e_{i}{}^{2}<0$, i.e., the manufacturer will not accept the contract. $\frac{1}{2}n\beta_{i_{0}}e_{i}{}^{2}$ is the negative effects when effort level reaches $e_{i}$ for type i manufacturers, where n represents the negative utility coefficient generated by the manufacturer’s efforts, and $\beta_{i_{0}}$ is the initial technical level parameter of type i manufacturer.

**Assumption** **5.** *The government’s incentive expenditure comes from the consumer’s taxes.* λ(λ>0) *indicates the shadow cost of public funds, and the actual expenditure of taxpayers is $T_{i}=\left(1+\lambda \right)t_{i}$.*

The main parameters involved in this paper are shown in Table 1.

## 3. Model Analysis

### 3.1. The Government’s Optimal Incentive Contract under Complete Information

With complete information, the government can accurately determine the type of manufacturer. It only needs to examine the optimal transfer payment and the optimal technological level of the manufacturer in the established contract. If a type *i* manufacturer chooses the contract $\left\{t_{i},\Delta \beta_{i}\right\}\left(i=H\;\mathrm{or}\;L\right)$, the government needs to solve the following problems:

(3) $$\begin{array}{l} \max W_{U}\left(e_{i}\right)=U_{S}(e_{i})+U_{C}(e_{i})-T_{i}\\ \left(IR\right)\;\pi_{i_{0}}\geq 0\\ e_{i}\geq 0,i=H\;\mathrm{or}\;L \end{array}$$

In Formula (3), $W_{U}\left(e_{i}\right)$ is the expected net utility of the government, i.e., the difference between the expected utility of the government and the actual expenditure. (IR) is the participation constraint, i.e., the manufacturer’s retention utility. The government motivates the manufacturer to innovate in technology, which is conducive to the long-term development of the manufacturer. Therefore, the government does not need to reserve utility for the manufacturer, i.e., $\pi_{i_{0}}=0$. By optimization, the following can be determined:

$$e_{i}{}^{*}=\frac{\left(va+b\right)k}{\left(1+\lambda \right)n\beta_{i_{0}}}\;t_{i}{}^{*}=\frac{1}{2}\frac{{\left(va+b\right)}^{2}k^{2}}{{\left(1+\lambda \right)}^{2}n\beta_{i_{0}}}\;\Delta \beta_{i}{}^{*}=\frac{\left(va+b\right)k^{2}}{\left(1+\lambda \right)n\beta_{i_{0}}}\;\pi_{i_{0}}=0\;\left(i=H\;\mathrm{or}\;L\right)$$

See Appendix A for the solution process.

**Corollary** **1.** *With complete information, the optimal contract* $\left\{t_{i}{}^{*},\Delta \beta_{i}{}^{*}\right\}\left(i=H\;\mathrm{or}\;L\right)$ *provided by the government to the manufacturer is:*

(4) $$\left\{\frac{1}{2}\frac{{\left(va+b\right)}^{2}k^{2}}{{\left(1+\lambda \right)}^{2}n\beta_{i_{j}}},\frac{\left(va+b\right)k^{2}}{\left(1+\lambda \right)n\beta_{i_{j}}}\right\}\;\left(i=H\;\mathrm{or}\;L,j\geq 0\right)$$

*where j represents phase j + 1 and* $\beta_{i_{j}}$ *represents the initial technical level of type i manufacturer in phase j + 1.*

With the complete information, the government can accurately know the initial technology level of the manufacturer in the first phase, as well as the technological upgrading level of the manufacturer after the end of the first phase. Therefore, the government in the second phase is facing the incentive armed with complete information. By analogy, the government can accurately know the technical level of the manufacturer before the incentive is offered. Therefore, with complete information, the government can achieve the ideal incentive in each period.

**Theorem** **2.** *In the government’s optimal incentive contract with complete information, the optimal transfer payment and the optimal effort level are positively correlated with the manufacturer’s initial technology level; the manufacturer cannot make a profit in the incentive project; when the same amount as the transfer payment is made, the government can get more expected net utility by motivating a high-tech manufacturer than a low-tech manufacturer.*

Corollary 1 quantitatively gives the optimal contract that the government should provide for the manufacturer with complete information. The contract can effectively promote the efforts of new energy vehicle manufacturers to improve their technology. Theorem 2 describes the relationship between the parameters in the optimal contract, pointing out that the government has encouraged high-tech manufacturers to obtain greater net benefits, providing a reference for government policy development.

### 3.2. Optimal Incentive Contract of the Government with Asymmetric Information

Symmetric information is the most ideal state for the government, but in reality, the government cannot observe the initial technology level of manufacturers. At this point, the government needs to identify the type of manufacturer and provide two kinds of contracts thereto at the same time: $\left\{t_{H},\Delta \beta_{H}\right\},\left\{t_{L},\Delta \beta_{L}\right\}$ The government needs to consider the following issues:

(5) $$\begin{array}{l} \max W_{U}{}^{A}=\alpha W_{U}\left(e_{H}\right)+\left(1-\alpha \right)W_{U}\left(e_{L}\right)\\ \left(IC-H\right)\;t_{H}-\frac{1}{2}n\beta_{H_{0}}e_{H}{}^{2}\geq t_{L}-\frac{1}{2}n\beta_{H_{0}}e_{L}{}^{2}\\ \left(IC-L\right)t_{L}-\frac{1}{2}n\beta_{L_{0}}e_{L}{}^{2}\geq t_{H}-\frac{1}{2}n\beta_{L_{0}}e_{H}{}^{2}\\ \left(IR-H\right)\;t_{H}-\frac{1}{2}n\beta_{H_{0}}e_{H}{}^{2}\geq 0\\ \left(IR-L\right)\;t_{L}-\frac{1}{2}n\beta_{L_{0}}e_{L}{}^{2}\geq 0 \end{array}$$

In Formula (5), since the government only knows the type distribution probability of the manufacturer, the objective function is to provide the expected net utility of incentive contracts to the two types of manufacturer. “*IC*” is the incentive compatibility constraint, which requires manufacturers to report their own technology type truthfully, and “*IR*” is individual rationality constraint, which guarantees that manufacturers will participate in the contract.

The following optimization will be solved:

First of all, the following can be obtained by $\frac{1}{2}n\beta_{L_{0}}e_{L}{}^{2}-\frac{1}{2}n\beta_{H_{0}}e_{L}{}^{2}=\frac{1}{2}ne_{L}{}^{2}\left(\beta_{L_{0}}-\beta_{H_{0}}\right)$ and $\beta_{L_{0}}>\beta_{H_{0}}>0$

(6) $$\frac{1}{2}n\beta_{L_{0}}e_{L}{}^{2}-\frac{1}{2}n\beta_{H_{0}}e_{L}{}^{2}\geq 0$$

That is to say, high-level manufacturers can always imitate the efforts of low-level manufacturers at lower costs, while the individual rational constraints of high-level manufacturers can be ignored.

The incentive compatibility constraints of low-level manufacturers are temporarily ignored, but will be verified later. Only the incentive compatibility constraints of high-level manufacturers and the individual rational constraints of low-level manufacturers are retained.

$$\begin{array}{l} t_{H}-\frac{1}{2}n\beta_{H_{0}}e_{H}{}^{2}\geq t_{L}-\frac{1}{2}n\beta_{H_{0}}e_{L}{}^{2}\\ t_{L}-\frac{1}{2}n\beta_{L_{0}}e_{L}{}^{2}\geq 0 \end{array}$$

The individual rationality constraint is indicated as $\begin{array}{l} \left(IR-H\right)\;\pi_{H_{0}}\geq 0\\ \left(IR-L\right)\;\pi_{L_{0}}\geq 0 \end{array}$. Rewrite the incentive compatibility of high-level manufacturers to obtain:

(7) $$t_{H}-\frac{1}{2}n\beta_{H_{0}}e_{H}{}^{2}\geq t_{L}-\frac{1}{2}n\beta_{H_{0}}e_{L}{}^{2}=t_{L}-\frac{1}{2}n\beta_{H_{0}}e_{H}{}^{2}+\frac{1}{2}n\beta_{H_{0}}e_{H}{}^{2}-\frac{1}{2}n\beta_{H_{0}}e_{L}{}^{2}$$

Formula (IR-L) and Formula (7) are optimal only when they are tight.

(8) $$\begin{array}{l} \max W_{U}{}^{A}=\alpha W_{U}\left(e_{H}\right)+\left(1-\alpha \right)W_{U}\left(e_{L}\right)\\ \;=\alpha \left(vake_{H}+bke_{H}-\left(1+\lambda \right)\frac{1}{2}n\beta_{H_{0}}e_{H}{}^{2}-\lambda \left[\frac{1}{2}ne_{L}{}^{2}\left(\beta_{L_{0}}-\beta_{H_{0}}\right)\right]\right)\\ +\left(1-\alpha \right)\left[vake_{L}+bke_{L}-\left(1+\lambda \right)\frac{1}{2}n\beta_{L_{0}}e_{L}{}^{2}\right] \end{array}$$

Set: $\frac{\partial W_{U}{}^{A}}{\partial e_{H}}=0$, $\frac{\partial W_{U}{}^{A}}{\partial e_{L}}=0$

Obtain:

Finally, the incentive compatibility constraints of low-level manufacturers are validated, and $e_{L}{}^{A*}$ are put into the substitution (IC-L), which is verified to satisfy the constraints.

**Corollary** **3.** *With asymmetric information, the optimal screening contract* $\left\{t_{i}{}^{A*},\Delta \beta_{i}{}^{A*}\right\}\left(i=H\;\mathrm{or}\;L\right)$ *provided by the government for the manufacturer is shown in Table 2.*

**Theorem** **4.** *With asymmetric information, the screening contract formulated by the government has the following characteristics:*

*(1)*  *Information symmetry does not affect the efforts of high-level manufacturers, but in the case of asymmetric information, high-level manufacturers can obtain information rent in incentive projects.*
*(2)*  *When the information is asymmetric, low-level manufacturers do not make enough efforts and cannot get information rent in the incentive project.*

**Theorem** **5.** *In incentive projects, the asymmetry of information reduces the expected net utility of the government.*

Corollary 3, and Theorems 4 and 5 show that due to the existence of asymmetric information, the government needs to design a screening contract to disclose the type of manufacturer. In this incentive period, compared with symmetric information, the high-level manufacturer can obtain information rent, so screening the contract is beneficial to the high-level manufacturer. While the low-level manufacturer makes insufficient efforts and has no information rent, the expected net utility of the government will reduce, so screening contracts are not ideal incentive contracts for low-level manufacturers and governments.

So far, the government has solved the problem of information asymmetry by screening the contract design, and knows exactly what type of manufacturer it belongs to. Therefore, every incentive period in the future can be designed armed with complete information about the optimal contract. So, we get:

**Corollary** **6.** *The optimal incentive contract of government with asymmetric information:*

$$\left\{ \begin{array}{l} \left\{t_{H}{}^{A*},\Delta \beta_{H}{}^{A*}\right\},\left\{t_{L}{}^{A*},\Delta \beta_{L}{}^{A*}\right\}\;\left(j=0\right)\\ \left\{\frac{1}{2}\frac{{\left(va+b\right)}^{2}k^{2}}{{\left(1+\lambda \right)}^{2}n\beta_{i_{j}}},\frac{\left(va+b\right)k^{2}}{\left(1+\lambda \right)n\beta_{i_{j}}}\right\}\;\left(i=H\;\mathrm{or}\;L,j\geq 1\right) \end{array}\right.$$

*where j represents phase j + 1 and* $\beta_{i_{j}}$ *represents the initial technical level of type i manufacturers in phase j + 1.*

Corollary 6 shows that with complete information, the government can achieve the most ideal incentive in each period; with asymmetric information, the government only needs to solve the problem of information asymmetry in the first incentive period through the design of a screening contract; then, the most ideal incentive with complete information can be achieved in each subsequent period.

Corollary 3 quantitatively gives the optimal contract that the government should provide in order to identify the type of manufacturer with asymmetric information. The contract can effectively identify the manufacturer type, and help the government solve the problem of information asymmetry; Theorems 4 and 5 and Corollary 6 describe the relationship between the parameters in the optimal contract, and point out that the government encourages high-tech manufacturers to obtain greater net benefits, which provides a reference for government policy formulation.

The existence of asymmetric information, on the one hand, will affect the expected net utility of the government, but on the other hand, will lead to information rent. Therefore, in the next section, an example will be used to analyze the impact of the relevant parameters on the government’s expected net utility and information rent.

## 4. Information Value

As shown above, asymmetric information has an impact on the government’s expected net utility, information rent, and the optimal effort level of low-level manufacturers. Therefore, this section uses an example to analyze the value of information.

In the optimal contract designed by the government with asymmetric information, the low-level manufacturer has no information rent, while the high-level manufacturer will get information rent. The information rent for high-level manufacturers is:

(9) $$R=\frac{1}{2}n{\left(\frac{\left(1-\alpha \right)\left[vak+bk\right]}{\lambda \alpha \left[n\left(\beta_{L_{0}}-\beta_{H_{0}}\right)\right]+\left(1-\alpha \right)\left(1+\lambda \right)n\beta_{L_{0}}}\right)}^{2}\left(\beta_{L_{0}}-\beta_{H_{0}}\right)$$

Set $\Delta_{\beta }=\beta_{L_{0}}-\beta_{H_{0}}$.

**Theorem** **7.** *The information rent of high-level manufacturers increases with the difference of the initial technology level between the high- and low-level manufacturers.*

Firstly, the influence of the distribution probability of high-level manufacturers on information value is considered. Under symmetrical information, the expected net utility of the government is as follows:

$$W_{U}\left(e_{i}{}^{*}\right)=\alpha \left(U_{S}(e_{H}{}^{*})+U_{C}(e_{H}{}^{*})-T(e_{H}{}^{*})\right)+\left(1-\alpha \right)\left(U_{S}(e_{L}{}^{*})+U_{C}(e_{L}{}^{*})-T(e_{L}{}^{*})\right)$$

With asymmetric information, the expected net utility of the government is as follows:

$$W_{U}\left(e_{i}{}^{A*}\right)=\alpha \left(U_{S}(e_{H}{}^{A*})+U_{C}(e_{H}{}^{A*})-T(e_{H}{}^{A*})\right)+\left(1-\alpha \right)\left(U_{S}(e_{L}{}^{A*})+U_{C}(e_{L}{}^{A*})-T(e_{L}{}^{A*})\right)$$

Because $e_{H}{}^{*}=e_{H}{}^{A*}$:

$$W_{U}\left(e_{i}{}^{A*}\right)=\alpha \left(U_{S}(e_{H}{}^{A*})+U_{C}(e_{H}{}^{A*})-T(e_{H}{}^{A*})\right)+\left(1-\alpha \right)\left(U_{S}(e_{L}{}^{A*})+U_{C}(e_{L}{}^{A*})-T(e_{L}{}^{A*})\right)$$

Set

$$W\left(e_{i}{}^{*}\right)-W\left(e_{i}{}^{A*}\right)=\Delta_{E},e_{L}{}^{*}-e_{L}{}^{A*}=\Delta_{e},v=0.5,a=2,b=1,k=2,n=0.5,\lambda =0.5,\beta_{L_{0}}=2,\Delta_{\beta }=1,\alpha \in \left(0,1\right).$$

Table 3 shows that the difference of the government’s expected net utility under symmetric and asymmetric information conditions increases first and then decreases with the increase of the distribution probability of high-level manufacturers. When the distribution probability of high-level manufacturers is minimal or maximal, the symmetry of information has little effect on the government’s expected net utility. However, when the distribution probability of the high-level manufacturer is great, the expected net utility of the government under symmetric and asymmetric information conditions is great, and the impact of information asymmetry on the expected net utility of the government is the smallest, i.e., it is the most beneficial to the government.

It may be concluded that:

Information rent decreases as the distribution probability of high-level manufacturers increases, indicating that few high-level manufacturers can get more information rent when there are incentives for the new energy automotive industry with multiple low-level manufacturers and few high-level manufacturers. Therefore, high-level manufacturers always want to lead low-level manufacturers in terms of their technology in order to obtain more information rent when information asymmetry occurs.

The difference between the optimal effort level of low-level manufacturers under symmetric and asymmetric information conditions increases with the increase of the distribution probability of high-level manufacturers, which indicates that the smaller the distribution probability of high-level manufacturers, the lower the effort levels of low-level manufacturers, due to information asymmetry, and the more advantageous the situation for low-level manufacturers.

In summary, in government incentive projects, the higher the probability of distribution of high-level manufacturers, the greater the expected net utility of the government, which is what the government wants to see. However, if the distribution probability of the high-level manufacturers is higher, the high-level manufacturers cannot obtain more information rent, and the low-level manufacturers cannot make full use of their efforts in incentive projects. At this time, it is not ideal for either high- or low-level manufacturers.

Secondly, we consider the impact of the initial technological level difference between high- and low-level manufacturers on information value. Combined with the example analysis, take $v=0.5,a=2,\;b=1,k=2,n=0.5,\lambda =0.5,\beta_{L_{0}}=2$, $\Delta_{\beta }\in (0,2)$, α=0.5.

From Table 4, the difference of the government’s expected net utility under symmetric and asymmetric information conditions increases with the increase of the initial technical level difference between high- and low-level manufacturers. The greater the initial technical level difference, the greater the impact of information asymmetry on the government’s expected net utility, and the smaller the initial technical level difference, i.e., the better the situation for the government.

Obtain:

Information rent increases with the difference of initial technology level between high- and low-level manufacturers, which indicates that the higher the initial technology level of high-level manufacturers is compared to that of low-level manufacturers, the more information rent the high-level manufacturers can obtain in incentives. Therefore, high-level manufacturers always want to be more technologically advanced than low-level manufacturers.

The difference of optimal effort levels between low- and high-level manufacturers under symmetrical and asymmetrical information conditions increases with the difference of initial technology levels between low- and high-level manufacturers, which indicates that the smaller the difference of initial technology level, the lower the efforts of low-level manufacturer caused by information asymmetry, which is preferable for low-level manufacturers. Low-level manufacturers should strive to narrow the technological gap with high-level manufacturers.

In summary, in government incentive projects, the greater the difference between the initial technology level of high- and low-level manufacturers, the greater the impact of asymmetric information on the expected net utility of the government. This is a situation that the government does not want to see. Low-level manufacturers cannot make full use of their efforts, which is also a situation that low-level manufacturers do not want to see. However, the greater the difference in initial technology level, the more information rent high-level manufacturers can get in incentive projects, which is the better for high-level manufacturers.

According to the analysis of Theorem 7 and Table 3 and Table 4, the smaller the gap of the initial technology levels between high- and low-level manufacturers in the new energy automotive industry, and the greater the probability of the existence of high-level manufacturers, the smaller the impact of information asymmetry in government incentive projects on the expected net utility of the government, and the better the development for the new energy automotive industry. Therefore, in the context of the emergence of new automobile companies, the technology level of new automobile companies is uneven. The government needs to consider whether to control technology.

## 5. Conclusions

In a realistic scenario, new energy automobile manufacturers can strive to improve their technology levels under the government’s incentive program. This paper studies how the government could design incentive contracts and motivate manufacturers to strive to improve their technology. The paper draws the following conclusions:

In an optimal incentive contract of a government with complete information, the optimal transfer payment and the optimal effort levels are positively correlated with the initial technical level of the manufacturer; the manufacturer cannot make a profit in the incentive project; the government can obtain more expected net utility by motivating high-level manufacturers than low-level manufacturers with an equal amount of transfer payment. In an optimal incentive contract for a government with asymmetric information, the optimal efforts of high-level manufacturers are consistent with those under symmetric information conditions, but under asymmetric information conditions, information rent can be obtained in the incentive project; the optimal efforts of low-level manufacturers are less than those under symmetric information conditions, and information rent cannot be obtained in the incentive project.

Compared with complete and asymmetric information conditions, the existence of asymmetric information conditions reduces the expected net utility of the government; the information rent increases with the increase in the difference between the initial technical level of high- and low-level manufacturers. This study obtained a quantitative incentive contract under full information conditions, and a quantitative screening contract under asymmetric information conditions, which provides an effective reference for the design of government subsidy contracts.

In light of government subsidies, most Chinese new energy vehicle manufacturers tend to be risk-neutral, so this paper assumes that both the government and the manufacturer are risk-neutral. However, some manufacturers are risk-seeking or risk-averse; this paper does not consider the risk preferences of manufacturers and governments in the incentive process, which is a limitation. We will further study this aspect in the future.

## Figures and Tables

**Table 1:** Main parameters involved in this paper.

Symbol	Definition
*e*	Efforts made by manufacturers to improve their technical level under government incentives
*β*	Manufacturer’s technical level parameters
*t*	Government incentives for manufacturers to transfer payments for technological innovation
*W*	Government expectation utility
*v*	Value coefficient of unit energy conservation and emission reduction for the government
λ	Shadow cost of public funds
*π*	Manufacturer’s retention utility in incentive projects
*α*	Probability of high-level manufacturers
*R*	Information rent
*a*, *b*, *k*, *n*	constant

Note: For other symbols in the paper, the asterisk indicates the optimal value under each condition; superscript A indicates asymmetric information; subscript H indicates a high technical level; and the subscript L indicates a low technical level.

**Table 2:** Optimal Government Decisions in Screening Contracts with Asymmetric Information.

i	H	L
$e_{i}{}^{A*}$	$\frac{\left(va+b\right)k}{\left(1+\lambda \right)n\beta_{H_{0}}}$	$\frac{\left(1-\alpha \right)\left[vak+bk\right]}{\lambda \alpha \left[n\left(\beta_{L_{0}}-\beta_{H_{0}}\right)\right]+\left(1-\alpha \right)\left(1+\lambda \right)n\beta_{L_{0}}}$
$t_{i}{}^{A*}$	$\frac{{\left(va+b\right)}^{2}k^{2}}{2{\left(1+\lambda \right)}^{2}n\beta_{H_{0}}}+\frac{1}{2}{\left(e_{L}{}^{A*}\right)}^{2}\left(\beta_{L_{0}}-\beta_{H_{0}}\right)$	$\frac{n\beta_{L_{0}}{\left(1-\alpha \right)}^{2}{\left[vak+bk\right]}^{2}}{2{\left\{\lambda \alpha \left[n\left(\beta_{L_{0}}-\beta_{H_{0}}\right)\right]+\left(1-\alpha \right)\left(1+\lambda \right)n\beta_{L_{0}}\right\}}^{2}}$
$\Delta \beta_{i}{}^{A*}$	$\frac{\left(va+b\right)k^{2}}{\left(1+\lambda \right)n\beta_{H_{0}}}$	$\frac{\left(1-\alpha \right)\left[vak+bk\right]k}{\lambda \alpha \left[n\left(\beta_{L_{0}}-\beta_{H_{0}}\right)\right]+\left(1-\alpha \right)\left(1+\lambda \right)n\beta_{L_{0}}}$
R	$\frac{1}{2}n{\left(e_{L}{}^{A*}\right)}^{2}\left(\beta_{L_{0}}-\beta_{H_{0}}\right)$	0

**Table 3:** Impact of Distribution Probability of High-Level Manufacturers on Information Value.

	1	2	3	4	5	6
α	0.001	0.200	0.400	0.600	0.800	0.999
$\Delta_{W}$	0.004	0.382	0.705	0.917	0.875	0.010
R	0.444	0.410	0.360	0.284	0.160	0.001
$\Delta_{e}$	0.001	0.107	0.267	0.533	1.067	2.651

**Table 4:** Impact of Initial Technical Differentiation on Information Value.

	1	2	3	4	5	6
$\Delta_{\beta }$	0.001	0.400	0.800	1.200	1.600	1.999
$\Delta_{W}$	0.003	0.605	1.103	1.481	1.518	2.165
*R*	0.002	0.625	1.107	1.481	1.773	1.999
$\Delta_{e}$	0.001	0.167	0.314	0.444	0.561	0.666

## Appendix A

Substituting $U_{S}(e_{i})=vake_{i}$, $U_{C}(e_{i})=bke_{i}$, $T_{i}=\left(1+\lambda \right)t_{i}$, $\pi_{i_{0}}=t_{i}-\frac{1}{2}n\beta_{i_{0}}e_{i}{}^{2}$ into Formula (3) can obtain:

$$\begin{array}{l} \max W_{U}\left(e_{i}\right)=vake_{i}+bke_{i}-\left(1+\lambda \right)t_{i}\\ \left(IR\right)\;t_{i}-\frac{1}{2}n\beta_{i_{0}}e_{i}{}^{2}\geq 0\\ e_{i}\geq 0,i=H\;\mathrm{or}\;L \end{array}$$

Let $t_{i}-\frac{1}{2}n\beta_{i_{0}}e_{i}{}^{2}=0$, that is $t_{i}=\frac{1}{2}n\beta_{i_{0}}e_{i}{}^{2}$, substituting it into the objective function to ge:

$$W_{U}\left(e_{i}\right)=vake_{i}+bke_{i}-\left(1+\lambda \right)\frac{1}{2}n\beta_{i_{0}}e_{i}{}^{2}$$

To find the derivative about $e_{i}$ for $W_{U}\left(e_{i}\right)$, $W_{U}{\left(e_{i}\right)}^{\prime }=vak+bk-\left(1+\lambda \right)n\beta_{i_{0}}e_{i}$.

Let $W_{U}{\left(e_{i}\right)}^{\prime }=0$ get $e_{i}{}^{*}=\frac{\left(va+b\right)k}{\left(1+\lambda \right)n\beta_{i_{0}}}$, and then:

$$t_{i}{}^{*}=\frac{1}{2}\frac{{\left(va+b\right)}^{2}k^{2}}{{\left(1+\lambda \right)}^{2}n\beta_{i_{0}}},\;\Delta \beta_{i}{}^{*}=\frac{\left(va+b\right)k^{2}}{\left(1+\lambda \right)n\beta_{i_{0}}},\;\pi_{i_{0}}=0,\;\left(i=H\;\mathrm{or}\;L\right).$$

## Appendix B

**Proof** **of** **Theorem** **2.** Under Optimal Contract, $t_{i}-\frac{1}{2}n\beta_{i_{0}}e_{i}{}^{2}=0$. Set $t_{i}$ to be a fixed value: $t_{i}=t_{1}$. Then, $\Delta \beta_{H_{1}}{}^{*}=k\sqrt{\frac{2t_{1}}{n\beta_{H_{0}}}}$ $\Delta \beta_{L_{1}}{}^{*}=k\sqrt{\frac{2t_{1}}{n\beta_{L_{0}}}}$, and then deduce to $\Delta \beta_{H_{1}}{}^{*}>\Delta \beta_{L_{1}}{}^{*}$, and $W_{U}{\left(e_{H_{1}}\right)}^{*}>W_{U}{\left(e_{L_{1}}\right)}^{*}$. Theorem 2 is proved.  □

## Appendix C

**Proof** **of** **Theorem** **4.** First of all, $e_{H}{}^{A*}=e_{H}{}^{*}$ $\pi_{H_{0}}=\frac{1}{2}n{\left(e_{L}{}^{A*}\right)}^{2}\left(\beta_{L_{0}}-\beta_{H_{0}}\right)$. Where, $\beta_{L_{0}}>\beta_{H_{0}}$. Therefore, $\pi_{H_{0}}>0$. (1) in Theorem 4 has been proved; when $e_{L}{}^{*}-e_{L}{}^{A*}=\frac{\left(va+b\right)k}{\left(1+\lambda \right)n\beta_{L_{0}}}-\frac{\left(1-\alpha \right)\left[vak+bk\right]k}{\lambda \alpha \left[n\left(\beta_{L_{0}}-\beta_{H_{0}}\right)\right]+\left(1-\alpha \right)\left(1+\lambda \right)n\beta_{L_{0}}}>0$, $\pi_{L_{0}}=0$. (2) in Theorem 4 has been proved; Finally, Theorem 4 has been proved.  □

## Appendix D

**Proof** **of** **Theorem** **5.** It proves that since the manufacturer may be high-tech or low-tech, it is discussed in two cases:

(i) When the motivated manufacturer is a high-level manufacturer, the transfer payment and the required technological upgrading of the government to the high-level manufacturer under symmetric information conditions are $t_{H}{}^{*}=\frac{{\left(va+b\right)}^{2}k^{2}}{2{\left(1+\lambda \right)}^{2}n\beta_{H_{0}}}$ and $\Delta \beta_{H}{}^{*}=\frac{\left(va+b\right)k^{2}}{\left(1+\lambda \right)n\beta_{H_{0}}}$ respectively; with asymmetric information, the transfer payment and the required technological upgrading of the government to the high-level manufacturer are $t_{H}{}^{A*}=\frac{{\left(va+b\right)}^{2}k^{2}}{2{\left(1+\lambda \right)}^{2}n\beta_{H_{0}}}+\frac{{\left(va+b\right)}^{2}k^{2}}{2{\left(1+\lambda \right)}^{2}n\beta_{H_{0}}}$ and $\Delta \beta_{H}{}^{A*}=\frac{\left(va+b\right)k^{2}}{\left(1+\lambda \right)n\beta_{H_{0}}}$ respectively; that is, $t_{H}{}^{*}<t_{H}{}^{A*}$, $\Delta \beta_{H}{}^{*}=\Delta \beta_{H}{}^{A*}$ and then $W_{U}{}^{*}>W_{U}{}^{A*}$;
(ii) When the motivated manufacturer is a low-level manufacturer, the principle of the proof process is the same as (i);

To sum up, Theorem 5 is proved.  □

## Appendix E

**Proof** **of** **Theorem** **7.** $\underset{\Delta \to 0}{\lim}I\left(\Delta_{\beta }\right)=0$, $\frac{\partial I\left(\Delta_{\beta }\right)}{\partial \Delta_{\beta }}>0\;\left(\beta_{H_{0}}>\Delta_{\beta }>0\right)$. Theorem 7 is proved.  □
